# The “Bringing into Cultivation” Phase of the Plant Domestication Process and Its Contributions to *In Situ* Conservation of Genetic Resources in Benin

**DOI:** 10.1100/2012/176939

**Published:** 2012-05-22

**Authors:** R. Vodouhè, A. Dansi

**Affiliations:** ^1^Bioversity International, Office of West and Central Africa, 08 BP 0931 Cotonou, Benin; ^2^Laboratory of Agricultural Biodiversity and Tropical Plant Breeding, Department of Genetics, Faculty of Sciences and Technology (FAST), University of Abomey-Calavi (UAC), 071BP28 Cotonou, Benin; ^3^Department of Crop Science (DCS), Crop, Aromatic and Medicinal Plant Biodiversity Research and Development Institute (IRDCAM), 071BP28 Cotonou, Benin

## Abstract

All over the world, plant domestication is continually being carried out by local communities to support their needs for food, fibre, medicine, building materials, etc. Using participatory rapid appraisal approach, 150 households were surveyed in 5 villages selected in five ethnic groups of Benin, to investigate the local communities' motivations for plant domestication and the contributions of this process to *in situ* conservation of genetic resources. The results indicated differences in plant domestication between agroecological zones and among ethnic groups. People in the humid zones give priority to herbs mainly for their leaves while those in dry area prefer trees mostly for their fruits. Local communities were motivated to undertake plant domestication for foods (80% of respondents), medicinal use (40% of respondents), income generation (20% of respondents) and cultural reasons (5% of respondents). 45% of the species recorded are still at early stage in domestication and only 2% are fully domesticated. Eleven factors related to the households surveyed and to the head of the household interviewed affect farmers' decision making in domesticating plant species. There is gender influence on the domestication: Women are keen in domesticating herbs while men give priority to trees.

## 1. Introduction

Plant domestication is the evolutionary process whereby a population of plants becomes accustomed to human provision and control [1]. For many authors [2, 3], domestication is generally considered to be the end-point of a continuum that starts with exploitation of wild plants, continues through cultivation of plants selected from the wild but not yet genetically different from wild plants (initial phase of bringing into cultivation), and ends with the adaptation to the agroecology through conscious or unconscious human morphological selection and hence genetic differences distinguishing the domesticated species from its wild progenitor. According to local communities, the collection of plants from the wild for cultivation on farm (fields or home gardens) is a common practice continually being carried out under diverse agroecosystems. Many varieties, landraces, and cultivars of plants have been developed through this process to meet human (and/or animal) demand for food, fibre, medicine, building materials, and so forth [4].

Throughout the world, the process of plant domestication has been either broadly analysed [5–9] or studied for species or group of species including acacias [10], yam [11, 12], tomatoes [13], barley [1], rice [4, 14], baobab [15], leafy vegetables [16], and fonio [17, 18]. These studies revealed the existence of different steps in the domestication process and highlighted that the practices used to highly vary with the species and the sociolinguistic groups across countries. Therefore, it is useful to document the process at country level.

This study aims to investigate plant domestication in different ethnic groups and agroecological zones of the Republic of Benin in order to

- document the species diversity, the domestication levels, and the use of the species under domestication;
- understand the motives of the domestication and the factors affecting farmers' decision making in domesticating plant species;
- analyse the gender influence on plant domestication.

## 2. Material and Methods

### 2.1. The Study Area

The Republic of Benin is situated in west Africa, between the latitudes 6°10′ N and 12°25′ N and longitudes 0°45′ E and 3°55′ E [19]. It covers a total land area of 112,622 km^2^ with a population estimated at about 7 millions [20]. The country is partitioned into 12 departments inhabited by 29 ethnic groups [19]. The south and the centre are relatively humid agroecological zones with two rainy seasons and mean annual rainfall of 1500 mm/year [19]. The north is situated in arid and semiarid agroecological zones characterized by unpredictable and irregular rainfall oscillating between 800 and 950 mm/year with only one rainy season. Mean annual temperatures range from 26 to 28°C and may exceptionally reach 35 to 40°C in the far northern localities [20, 21]. The country has about 2,807 plant species [21]. Vegetation types are semideciduous forest (south), woodland and savannah woodland (centre east and northeast), dry semideciduous forest (centre west and south of northwest), and tree and shrub savannahs (far north).

### 2.2. Site Selection and Survey

**Figure 1:**
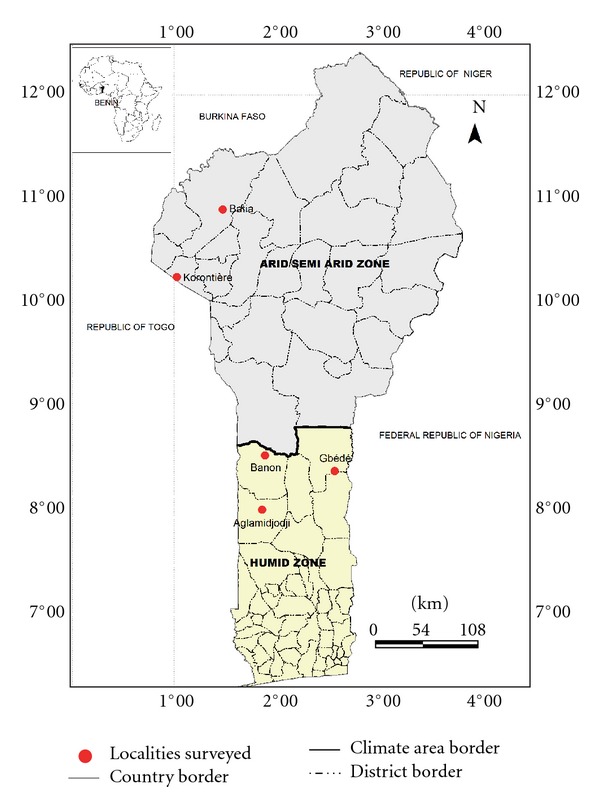
Benin map showing the location of the surveyed sites.

For the study, five villages (Aglamidjodji, Banon, Batia, Gbédé, and Korontière) were selected in the two contrasting agroecological zones of the country (Figure 1). Aglamidjodji, Banon, and Gbédé are located in the central region of Benin (humid zone), while Batia and Korontière are in the north (arid zone). In term of the vegetation type, Aglamidjodji and Korontière are entirely degraded; Banon and Gbédé are forested, while Batia is located in a savannah zone (Pendjari Park; Figure 1). Aglamidjodji, Banon, Batia, and Gbédé are inhabited, respectively, by the ethnic groups Mahi, Nago-Fè, Gourmanché, and Nago-Tchabè. Korontière is shared by two ethnic groups: the Ditamari (local and dominant) and the Lamba (originated from the Republic of Togo and in minority).

Data were collected during expeditions from the different sites through the application of participatory research appraisal tools and techniques such as direct observation, group discussions, individual interviews, and field visits using a questionnaire [16]. Interviews were conducted with the help of translators from each area. In each site, local farmers' organizations were involved in the study to facilitate the organization of group meetings (details of the research objectives were presented to the farmers, and general discussion was held on the steps of the plant domestication process) and assist in the data collection at household level.

 In each village, 30 households (total of 150 for the study zone) were randomly selected using the transect method described by Dansi et al. [16]. At household level, interview was conducted only with the head of family and his wife. However, in case of polygamy, all wives were involved in the discussions taking into consideration key roles played by women in plant domestication and biodiversity management and conservation on farm [22–25]. During each interview, sociodemographic data of the surveyed household (size, total area available, total area cultivated, number of crops practiced, area occupied by the major crops, number of food shortages experienced during the last ten years) and of its head (age, number of wives, number of the social groups to which he belongs, education level, age of his wife or first wife when many) were first collected. Then, the household head and his wife were asked to list (vernacular name) the species being domesticated by their household.

Field visits were conducted to see and document the listed species in their natural habitats (bushes, shallows) or where they are being cultivated (home gardens, cultivated fields). On each species inventoried, information recorded through discussions were related to status (wild, cultivated), life form (tree, shrub, and herb), habitat, part of the plant used and season of availability, importance (food, nutrition, medicinal values, etc.), reasons for domestication, and person (husband or his wife; gender issue) responsible for its domestication. Scientific names were determined by the plant taxonomist of the research team using the Analytic Flora of Benin [21], and pictures were taken for report.

Different steps exist in the bringing into cultivation phase of the plant domestication process. For each species, the level reached in this phase was determined and quoted using a seven-step model modified following Dansi et al. [16] and described as follows.

**Step 1**

Species entirely wild and collected only when needed.

**Step 2**

Wild species maintained in the fields when found during land preparation (clearance, burning, and weeding) due to its proved utility and regular need, its scarcity around habitations, and the difficulties for getting it on time, in quality and in quantity. These preserved plants are subject to regular observations for the understanding of their reproductive biology.

**Step 3**

Farmers start paying more attention to the preserved plants (weeding, protection against herbivorous) for their survival and their normal growth. A sort of ownership on the plants start.

**Step 4**

The reproductive biology of the species is known, and multiplication and cultivation of the species in the home gardens or in selected parts of cultivated fields are undertaken by farmers or healers. At this stage, farmers tend to conduct diverse experiments (date of planting, sowing or planting density, pest and diseases management, etc.) in order to master mass production of the species in the future. The ownership on the plant is more rigorous.

**Step 5**

The species is cultivated and harvested using traditional practices.

**Step 6**

To improve the quality of the product, farmers adopt specific criteria to select plants that better satisfied people needs. The best cultivars/plants (good grain/fruit quality, resistant/tolerance to diseases and pests) are known, and technical package is adopted for their development and multiplication. At this stage, access to market is considered and some species benefit from traditional postharvest technologies (method for processing, cooking or conservation, etc.) to meet consumers' needs.

**Step 7**

Selection initiatives continue with cooking qualities, protection against pests, and diseases in cultivation and storage. Income generation is more clearly taken care of: market demands (quantity and quality) are also taken into account, and species varieties that meet consumers' preferences are selected and produced.

### 2.3. Data Analysis

Data were analysed through descriptive statistics (frequencies, percentages, means, etc.) in order to generate summaries and tables at different (villages, ethnic groups, households) levels. To compare the mean numbers of species in domestication recorded per household between ethnic groups or agroecological zones, the nonparametric tests of Wilcoxon and of Kruskal-Wallis were computed using SAS [26]. To analyse the relationships between villages in term of species in domestication, villages surveyed were considered as individuals and the plant species under domestication as variables and scored, for each village, as 1 when present or 0 if not. Using this methodology, 69 variables (corresponding to the species inventoried) were created and a binary matrix was compiled. Pairwise distances between villages were computed by NTSYS-pc 2.2 [27], using Jaccard coefficient of similarity [28]. Similarity matrix was used to design a dendrogram using UPGMA cluster analysis [29, 30]. The same process was used to examine the distribution of the species with regards to their levels of domestication and habitats. Here, the 69 species inventoried were still considered as individuals and the different domestication levels and habitats recorded as variables and also scored as 1 when present or 0 when absent. The binary matrix compiled was used to perform a principal coordinate analysis (PCA) and generate a dendrogram as described above using the same software packages. Spearman coefficient of correlation was calculated using SAS statistical package [26] to test the influence of six variables related to the households surveyed (size, number of crops practiced, total area available, total area cultivated, total area occupied by the major crops, number of food shortages experienced the last ten years) and of five parameters linked to the head of the household interviewed (age, education level, number of wives, age of the first wife, number of the social groups to which he belongs) on the household decision making with regard to the number of species to domesticate. 

## 3. Results

### 3.1. Sociodemographic Profile of the Households Surveyed

The size of the households surveyed varied from 1 to 40 with 9 on average. The maximum size (40) was obtained at Banon and the minimum (1) at Aglamidjodji and at Batia. Among the 150 respondents, 25.34% were women and 74.66% were men; 51.66% have never been to school, 30.83% went to primary school, and 17.51% attended secondary school. The average age of the respondents was 40 years (minimum 20 years; maximum 75 years). The majority (79.16%) of the men respondents had one to two wives. Most of the respondents (71%) did not belong to any farmers' association (group), 22% belong to one, two, three, or four groups, and a very few number (7%) are members of 5 to 6 groups. 

### 3.2. Diversity of the Species under Domestication

Throughout the five villages surveyed, a great diversity of plant species under domestication was found. A total of 69 species belonging to 62 genera and 40 families (Table 1) were inventoried and documented. Among the 40 families, the five most important were the Leguminosae-Caesalpinioideae (7 species), the Lamiaceae (5 species), the Asteraceae (4 species), the Moraceae (3 species), the Bombacaceae (3 species), and the Asclepiadaceous (3 species). The remaining families (34) have only one to two species. For these 69 species inventoried, 138 vernacular names (Table 1) were recorded. They vary from place to place and sometime within the same ethnic group (Table 1). Per village, the total number of species under domestication inventoried varies from 18 (Aglamindjodji) to 32 (Banon) with 24 species on average per village (Table 2). The species found consisted of 19 trees (27.53%), 11 shrubs (16%) and 39 (56.47%) erect, creeping or climbing herbs (Table 1). A higher proportion of trees was observed in the northern region (Korontière and Batia) in comparison to the southern zone (Table 2).

**Figure 2:**
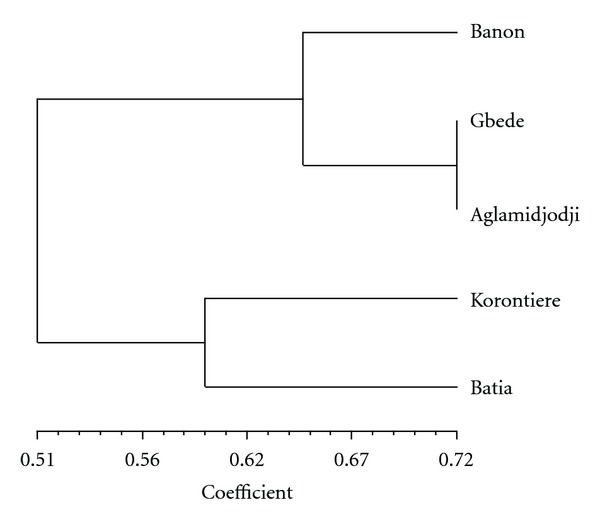
UPGMA dendrogram based on Jaccard coefficient of similarity showing the grouping of the villages.

Geographic distribution of the species inventoried showed high variability (Table 1). Some species such as *Adansonia digitata*, *Parkia biglobosa*, *Sesamum radiatum*, *Vitellaria paradoxa, *and *Vitex doniana* were found under domestication in all the villages surveyed, while many others like *Celosia trigyna*, *Cleome ciliate,* and *Lippia multiflora *were restricted to only one or two sites (Table 1). The great majority (50 to 71%) of the species was found in forests or fallows (Table 2). Only a few numbers were found in cultivated fields or in the home gardens. The mean number of species found under domestication per household significantly (*P* = 0.0002) varied between agroecological zones and among ethnic groups, but no significant difference was obtained between savannah and forest zones. In the humid zone, the mean number of species per household recorded was 8, while, in the arid zone, it was 5. At 30% of similarity level, the dendrogram constructed to analyse the relationships between surveyed villages in term of species under domestication led to two groups, namely, G1 and G2 (Figure 2): G1 gathers Batia and Korontière, the two villages of the north, while G2 assembles the three villages of the centre (Aglamidjodji, Banon, and Gbédé).

In all the villages surveyed, most of the species (61.90 to 77.77%) under domestication were well known to the local communities at both taxonomical and biological (growth, ecological requirements, reproduction) levels (Table 3). Among the species inventoried, three were reported as under threat due to over exploitation by people. These were *Caesalpinea bonduc*, *Launeae taraxacifolia,* and *L. multiflora*.

### 3.3. Availability and Utilisation of the Species

**Figure 3:**
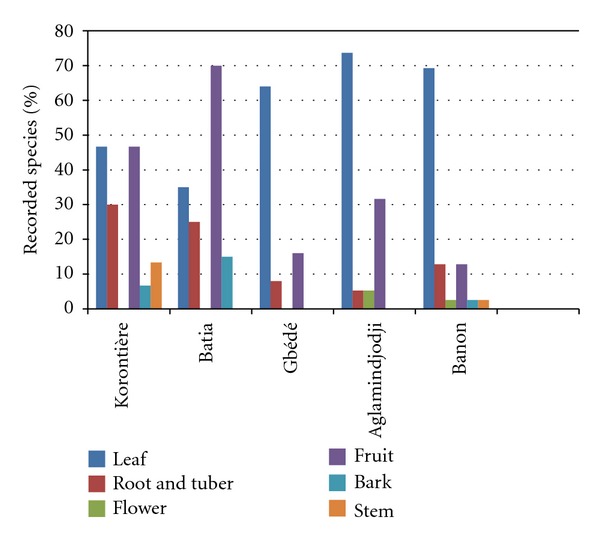
Relative importance of the species under domestication with regard to their organs used across villages.

Three groups of plant species were found when considering the availability period of the part of the plant used (Figure 3). The first group is made of species available for use only in rainy season; the second contains those used only in dry season, while the third group refers to species available the whole year. At Aglamidjodji, Banon, Gbédé, and Korontière, species of the first group were the most important followed by those of group 3. At Batia, the proportion of the species in group 2 outstrips the ones in group 3.

The organs (leaves, fruits, bark, roots, tuber, and flowers) of the different species inventoried used by the local communities vary considerably with the species and ethnic groups (Table 1). At Batia (Gourmantché zone), the species domesticated for their fruits are the most important followed by those domesticated for their leaves (Figure 3). In the other four villages (Aglamidjodji, Banon, Gbédé, and Korontière), the situation is opposite: species from which leaves are the most useful parts were the most numerous followed by those used for their fruits (Figure 3). Out of the 69 species inventoried, fourteen were domesticated only for medicinal purposes, three (*Cochlospermum tinctorium*, *L. taraxacifolia* and *L. multiflora*) were typically nutraceutical (as they have medicinal properties beside their nutritional value), and the others (52 in total) are used for food or medicine depending on the part of the plant considered (Table 1).

### 3.4. Domestication Levels of the Species

The domestication levels recorded for the species inventoried vary from 0 to 5. The number of species decreased with the domestication level. The majority of these (31 species, 45%) was found at Step 1 in all the villages where they have been signalled, and only one species (*Dioscorea praehensilis*) was found at Step 6 (Table 4). For most of the species (38 in total, Table 4) other than those found at Step 1 in all the villages, the domestication level is not consistent from one village to the other (Table 4). *S. radiatum,* for example, is at Step 1 at Korontière, Step 2 at Gbédé, Step 3 at Aglamidjodji, and Banon and Step 5 at Batia (Table 4).

**Figure 4:**
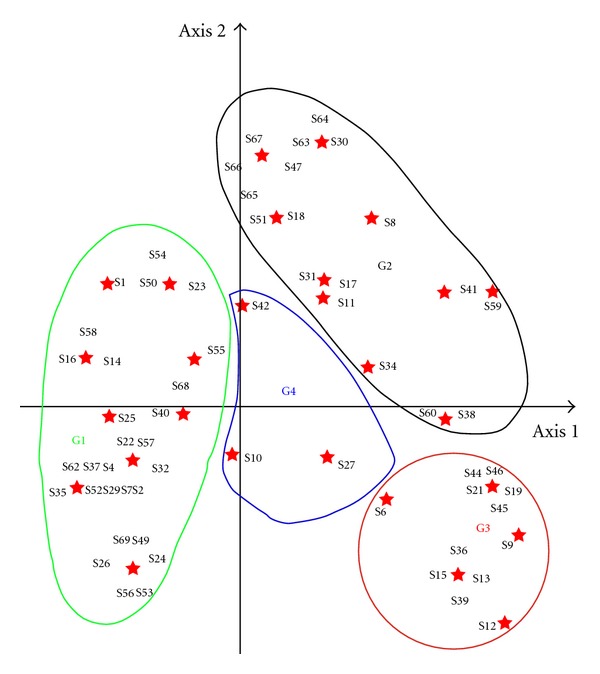
Principal coordinate analysis showing grouping of the species in relation to habitat and domestication levels. Species codes are those used in Table 1.

The principal coordinate analysis carried out to analyse the relationships among species in terms of habitat and domestication levels led to four groups, namely, G1, G2, G3 and G4 (Figure 4).

- G1 gathers the wild species which naturally occur in the forests, savannahs and fallows and which are at Step 1.
- G2 is the group of the species spared in the fields when found during land preparation and which received no or very little management attention from farmers for their survival (species found at Step 2 or 3).
- G3 assembles all the species found at Step 4 of the overall domestication process. It is the group of the species under cultivation in home gardens or in specific parts of cultivated fields.
- G4 pulls together the cultivated species found at Step 5 (*Calotropis procera*/S10; *L. multiflora*/S42) and at Step 6 (D. praehensilis/S27).

**Figure 5:**
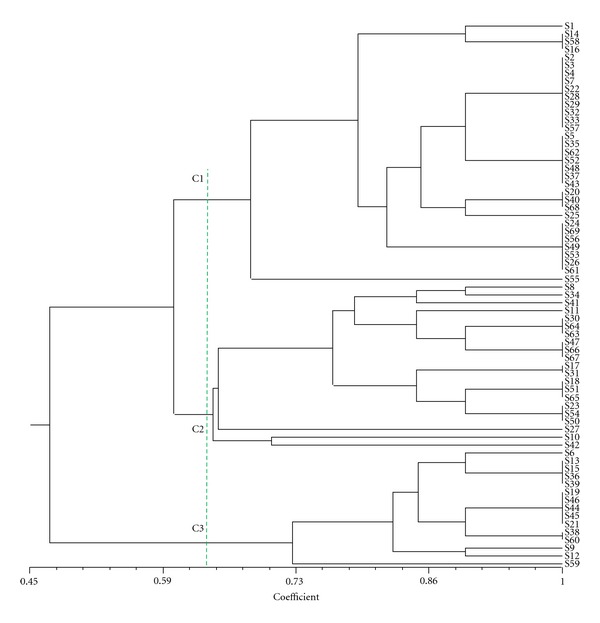
Dendrogram showing the classification of the species base on their habitat and their domestication levels.

At 60% of similarity, the dendrogram (Figure 5) of the UPGMA cluster analysis performed on the same data revealed tree classes (C1, C2, C3) of which two (C1 and C2) correspond, respectively, to G1 and G2, while the third one (C3) is G3 and G4 pulled together.

### 3.5. Motivations behind the Plant Domestication

According to farmers, the domestication of a plant starts, when its usefulness is proved, its demand is confirmed and regular, its availability around dwellings is seriously decreasing and when getting the desired quantity on time for use becomes problematic. They reported that plant domestication is generally done by simple curiosity or for dietary, medicinal, economic, or cultural reasons. Among these reasons, the most important is food security (50.85% of respondents) followed by medicinal use (30.5% of respondents), economic reasons (14.41% of respondents), and cultural reasons (4.24% of respondents).

In fact, many of the species recorded are sold in the markets and their annual contribution to household income generation and poverty reduction is appreciable (Table 5). A comparison between economic values and domestication levels of twelve species (Table 5) revealed that species such as *Ceratotheca sesamoides*, *C. tinctorium*, *L. taraxacifolia, *and *P. biglobosa* although having a relatively high economic value (in the rural areas surveyed), are still at very low domestication levels. *C. tinctorium,* for example, is still at Step 1of the domestication process, while its root (dried and grinded to a powder) is highly valued as nutraceutical vegetable (treatment of malaria, diabetes) in the northern regions of Benin. One species (*Agelanthus dodoneifolius*) was domesticated only for cultural reasons. In Lamba ethnic zone, one believes that it protects houses against evil spirits. Several factors affect farmers' decision making in domesticating plants. A correlation analysis revealed that among eleven (11) parameters related to the households surveyed and to the head of the household interviewed, eight are significantly correlated (*P* < 0.0001) with the number of species domesticated per household either positively (size of the household, age of the head of the household, age of the household wife, total area available, total area cultivated, area occupied by the major crops) or negatively (education level of the head of the household, number of food shortages experienced during the last ten years) while three (Number of wives, number of the social groups, number of crops practiced) showed no significant correlation.

### 3.6. Gender and Plant Domestication

The number of species found under domestication varied according to the gender (Table 6). Out of the 69 species recorded throughout the five villages surveyed, 31 (44.92%) were found under domestication with only women, 18 (26.08%) with only men, and 20 (28.98%) with both men and women. Some differences were observed between ethnic zones (Table 6). Hence, in the cultural areas Nago and Mahi (central Benin), the number of species being domesticated by women (50 to 55.55% of the total) is higher than the ones under the control of men. Contrary to Nago and Mahi ethnic groups, in the Gourmantché, Ditamari, and Lamba ethnic groups in northern Benin, men domesticated more species (42.85 to 59.25% of the total) than women. The classification of the species recorded according to both gender and use revealed that species being domesticated by women were basically leafy vegetables while those linked to men were essentially fruit species (Table 6) and the species being domesticated by both men and women were medicinal plants.

## 4. Discussion

### 4.1. Diversity, Availability, and Utilisation of the Species

The process of plant domestication is very active in the rural areas of Benin. The great diversity of the species under domestication recorded in this study is a tangible proof. These results are in support of those reported earlier on yam [11, 12, 31] and on traditional leafy vegetables in Benin [16]. For the 69 species inventoried, 138 vernacular names were recorded. Many names (one to five) were known for each species, and these vary among and within ethnic areas (Table 1). In the study of folk nomenclature in plant, such variation is now well known and documented [16, 32, 33]. The higher numbers of species under domestication were found in the forest zones and most of species recorded (56.47%) were herbaceous. Herbaceous are annual and are not available at the same place all the years and searching for an important wild herb species within the forest when needed is not secure (frequent snakebites, risks of lost). The species inventoried do not have the same ecogeographical distributions, and moreover the indigenous knowledge related to the utilization of the species varies from one area to the other. One understands, therefore, why some species were found under domestication in all the villages surveyed while many others were restricted to only one or two sites.

The ecogeographical consideration also remains the main justification of the partition (based on the species found under domestication) of the five villages surveyed into two clusters corresponding to the arid zone of the north and to the humid zone of the south. The communities interviewed have a good knowledge of the status of the plant species they are domesticating. They reported tree species (*C. bonduc*, *L. taraxacifolia,* and *L. multiflora*) under threat due to overexploitation by people. This is true for *L. taraxacifolia* following Dansi et al. [16] and also for *C. bonduc* and *L. multiflora*, which are even already in the Benin red list of threatened species [20]. The great majority of the species was used for food and/or medicine, the two most important vital needs of human being. Similar results were reported by Hildebrand [34] in southwest Ethiopia and by Casas et al. [6] in Mesoamerica. In all the villages surveyed apart from Batia, most of the species are being domesticated for their leaves besides available for use mainly in rainy season. This result is expected as most of the species domesticated for their leaves are leafy vegetables of daily used [16]. At Batia, bordering village of the national park of Pendjari inhabited by the Gourmantché, fruit species are most numerous and the plants whose useful parts are available only in dry season were preferred. The richness of savannah woodland in fruit trees and preference for fruit species by the ethnic groups living in the area may be the explanations of this finding.

### 4.2. Motivations behind the Plant Domestication and Domestication Levels

Farmers reported that plant domestication seeks to bring out the maximum human benefit within a species. It is generally done for dietary, medicinal, economic, and cultural reasons or by simple curiosity. This result is in agreement with those reported by Hildebrand [34] and Casas et al. [6]. Not surprisingly, the number of species domesticated per household is affected by several factors dominated by the education level of the head of the household and the number of food shortages experienced the last ten years. The negative influence noted for the first factor follows the actual general tendency by intellectuals to abandon traditional practices. On the other hand, the negative correlation observed with the number of food shortages experienced the last ten years was unexpected and could be tentatively explained as follow: a species being domesticated for food purposes is rarely cultivated or present on a large area in a short period of time. Consequently, it cannot produce sufficient quantity of food needed to meet the requirements of the households which are generally important. It is therefore normal that the more a household experienced food shortages, the more they will abandon domestication in favour of a more strengthened production of staple crops (cereals, root, and tubers, etc.).

Most of the species were found at low levels of domestication apart from yam where domestication was well studied and understood at both ethnobotanical and molecular levels [11, 12, 31, 35]. Normally species with high economic value should be prioritised for domestication by the households. Unfortunately, *C. tinctorium*, *L. taraxacifolia, *and *P. biglobosa* although having a relatively high economic value are still at very low domestication levels. For the farmers interviewed, *C. tinctorium* is still plenty in the wild and not very far from the villages; therefore, there is no urgent need to cultivate it. On the other hand, collecting its roots from the bush is laborious and grinding them later on into powder after drying is very difficult. They recognize however that *L. taraxacifolia *is becoming rare, but its domestication cannot go further than the “let standing” (practices directed to maintain within human-made environments useful plants that occurred in those areas before the environments were transformed by humans) described by several authors [6, 36–39] due to its reproductive biology (rapid loss of viability of the seeds during storage) not yet understood. For *P. biglobosa,* the reasons are not clear enough. The long time needed for the plant to start producing fruits could be the major handicap. Shortening the growth cycles for most fruit trees will facilitate their domestication process.

The results of the multivariate analysis (PCA and Cluster analysis) indicates that the seven steps (Step 1 to 7) initially defined in the domestication process could be visibly reorganized into three. The first one corresponds to Step 1, the second to the combination of Steps 2 and 3, and the third one associates Steps 4 to 7. These three newly defined steps correspond to the three different practices (systematic gathering, let standing, encouraging growing) defined by many authors [6, 40–44].

### 4.3. Gender Issue and Role of Domestication in Conserving Plant Diversity on Farmlands

Variation was noted on the number of species found under domestication according to the gender. In the south, female-headed households domesticate more species than male-headed households. In the north, the opposite situation was observed. In both cases, species being domesticated by women were basically leafy vegetables and medicinal plants while those under the control of men were mainly fruits. The cultural division of tasks at household level generally devotes women to food preparation and children care taking, and men to hunting and farming. Richness of savannah woodland in wild fruit trees and the fruit harvest which is typically men activity because of the physical skill and energy it requires could be a comprehensive explanation of these results which are in agreement with those published by Msuya et al. [9] in Tanzania.

The great diversity (69 species) of plant recorded indicates that domestication is a traditional practice for conserving biodiversity. Domestication contributes to increasing plant genetic diversity and to conservation on farm of the agricultural biodiversity. It is a dynamic system which links genetic diversity development, use, and conservation. This observation is in agreement with publications of many scientists [9, 45–50] who studied plant domestication in many parts of the world. Many species that are on the red list of Benin, threatened species like *C. bonduc,* would have completely disappeared, if they have not been domesticated by local communities. Similar results were reported in Cameroon and Madagascar, where domestication of *Prunus africana* Hook. f. has protected the species against extinction because of excessive bark harvesting for export for medicinal use [48, 51].

## 5. Conclusion

This study showed that domestication is actively being carried out in the rural areas of Benin and appears as a one of the most appropriate practices for developing the diversity, increasing its use and conserving agricultural biodiversity *in situ*. The process follows different steps which can be deliberately organised into three, four, or six steps. The results highlighted the role that gender (men and women) plays in plant domestication and revealed that food security and health, two vital needs of human being, are the main motives behind adoption and cultivation of wild species. Thanks to local communities' efforts, experiences, and innovations, plant genetic diversity is being developed, preserved, and sustainably used. Unfortunately, several factors limit full success of farmers' initiatives: limited knowledge of plant reproductive biology, plant diseases and pests' complex, climate variability and its impact on biodiversity, and so forth. Scientific investigations on major constraints to plant domestication are needed. We recommend that multidisciplinary research focusing on individual plant species (leafy vegetables, herbs, fruits, etc.) be conducted to better understand the influence of the domestication on the evolution of the species. Further baseline studies are needed on the uses and values of the species under domestication by the local communities throughout west Africa.

## Figures and Tables

**Table 1:** Diversity, vernacular names, and utilisation of the species under domestication across ethnic groups.

Number	Scientific names	Family	Vernacular name	Part of the plant used
1	*Adansonia digitata *	Bombacaceae	Otché (Fè, Nago), Télou (Lamba), Zouzon (Mahi), Boutouobou (Gourmantché)	Gourmanthé, Nago, Fè (Fruits and Leaves); Lamba (fruits)
2	*Agelanthus dodoneifolius*	Loranthaceae	Ayapou (Lamba)	Lamba (bark)
3	*Annona senegalensis*	Annonaceae	Alilou (Lamba)	Lamba (Leaves, fruits)
4	*Anogeissus leiocarpus*	Combretaceae	Kolou (Lamba)	Lamba (bark)
5	*Balanites aegyptiaca*	Balanitaceae	Boukpanwounkpôhôbou (Gourmantché)	Gourmantché (fruits)
6	*Bixa orellana*	Bixaceae	Timinti-éssô (Fè)	Fè (fruits)
7	*Blighia sapinda *	Sapidaceae	N'tchin (Nago)	Nago (fruits)
8	*Bombax costatum*	Bombacaceae	Kpahoudèhouin (Mahi), Houlou (Lamba)	Mahi, Lamba (Leaves)
9	*Caesalpinia bonduc *	Fabaceae-caesalpinioideae	Adjikoun (Mahi), Ogrounfè (Nago), Fèo (Fè)	Fè (Leaves, roots, seeds), Tchabè (Roots), Mahi (Root,
10	*Calotropis procera *	Asclepiadaceae	Touloukou (Lamba)	Lamba (Leaves)
11	*Ceiba pentandra*	Bombacaceae	Ogoun Fè (Fè)	Fè (Leaves)
12	*Celosia argentea*	Amaranthaceae	Tchôkôyôkôtô (Nago), Sôman (Mahi)	Nago, Mahi (Leaves)
13	*Celosia trigyna*	Amaranthaceae	Adjèmanwofô (Nago, Fè),	Nago, Mahi (Leaves)
14	*Ceratotheca sesamoides *	Pedaliaceae	Agbôssou (Mahi), Koumonkoun (Fè), Idjabô (Nago), Assoworou (Lamba)	Mahi, Fè, Gourmantché, Nago, Lamba (Leaves)
15	*Corchorus tridens*	Tiliaceae	Ountcho (Nago)	Nago (Leaves)
16	*Cissus populnea*	Vitaceae	Tchôkougbôlô (Fè), Kpôgôlô (Nago), Anyar (Lamba)	Fè, Nago, Lamba (roots)
17	*Clausena anisata*	Rutaceae	Oroukôgbo (Fè)	Fè (Leaves and roots)
18	*Cleome ciliata *	Capparaceae	Aiya (Mahi)	Mahi (Leaves)
19	*Cleome gynandra *	Capparaceae	Akaya (Nago)	Nago (Leaves)
20	*Cochlospermum tinctorium*	Cochlospermaceae	Boussôrôbou (Gourmantché)	Gourmanthé (Roots)
21	*Crassocephalum rubens*	Asteraceae	Akôgbo (Mahi), Gboolo (Nago, Fè)	Fè, Nago, Mahi (Leaves)
22	*Cymbopogon giganteus*	Poaceae	Kpalman mihou (Lamba)	Lamba (Leaves)
23	*Detarium microcarpum *	Leguminosae	Kpôr (Lamba), Bounankpôhôbou (Gourmantché)	Gourmantché, Lamba (Roots, fruits)
24	*Dichrostachys cinerea*	Leguminosae	Nanha sèhô (Lamba)	Lamba (Roots)
25	*Diospyros mespiliformis*	Ebenaceae	Ankalé (Lamba), Bougaabou (Gourmantché)	Lamba, Gourmantché (fruits)
26	*Dioscorea abyssinica*	Dioscoreaceae	Koudjabouwoungou (Gourmantché)	Gourmantché (Tuber)
27	*Dioscorea praehensilis *	Dioscoreaceae	Ichou (Fè)	Fè (Tuber)
28	*Echinops longifolius *	Asteraceae	Koumantchaintchain (Wama)	Wama (Roots)
29	*Eriosema pellegrinii*	Leguminosae	Kassimintê (Wama)	Wama (Roots)
30	*Ficus abutilifolia *	Moraceae	Agbèdè (Fè), Okpoto (Nago)	Fè, Nago (Leaves)
31	*Ficus ingens*	Moraceae	Boukankanbou (Gourmantché)	Gourmantché (Leaves)
32	*Ficus sycomorus*	Moraceae	Oukankanmou (Gnindé)	Gnindé (Leaves)
33	*Gardenia erubescens*	Rubiaceae	Bounansôôbou (Gourmantché), kaou (Lamba)	Gourmantché (Fruits), Lamba (Fruits, stem)
34	*Haumaniastrum caeruleum*	Lamiaceae	Atingbinnintingbin (Fè)	Fè (Leaves)
35	*Heteropteris leona*	Malpigluaceae	Nansikôr (Lamba)	Lamba (Leaves and Roots)
36	*Hibiscus sabdariffa *	Malvaceae	Kpakpala (Nago), Kpakpa (Fè)	Fè, Nago (Leaves)
37	*Indigofera bracteolata*	leguminosae	Tikouyè ogoutè (Gnindé)	Gnindé (Leaves and roots)
38	*Justicia tenella*	Acanthaceae	Djagou-djagou (Fè)	Fè (Leaves)
39	*Lagenaria siceraria *	Cucurbitaceae	kaka (Nago)	Nago (Leaves)
40	*Lannea microcarpa*	Anacardiaceae	Bougbantchabou (Gourmantché)	Gourmantché (fruits)
41	*Launeae taraxacifolia*	Asteraceae	Odôdô (Nago, Fè), Gnantotoé (Mahi)	Fè, Nago, Mahi (Leaves)
42	*Lippia multiflora*	Verbenaceae	Aglaala (Mahi), Tchaga (Fè)	Fè, Mahi (Leaves, flowers)
43	*Momordica charantia*	Cucurbitaceae	Tchaati (Fè), Gnissikin (Mahi)	Fè, Mahi (Leaves)
44	*Ocimum americanum*	Lamiaceae	Ofin (Fè)	Fè (Leaves)
45	*Ocimum basilicum*	Lamiaceae	Ounkpèhoun (Fè), Gbogbotyin (Nago), Hissin-hissin (Mahi)	Nago (Leaves)
46	*Ocimum gratissimum*	Lamiaceae	Simonba (Fè), Kioyo (Mahi)	Fè, Mahi (Leaves)
47	*Parkia biglobosa *	Leguminosae	Ayoya (Mahi), Ougba (Nago), Igba (Fè), Boudoubou (Gourmantché), S'lou (Lamba)	Mahi, Fè, Nago, Lamba (fruits); Gourmantché (Fruits, Bark)
48	*Pergularia daemia *	Asclepiadaceae	Agbonfoun-foun (Fè)	Fè (Leaves)
49	*Phyllanthus muellenianus*	Euphorbiaceae	Akanmankogou (Mahi)	Mahi (Leaves)
50	*Piliostigma thonningii *	Leguminosae	Wôkou (Lamba)	Lamba (Leaves, Roots)
51	*Platostoma africanum *	Lamiaceae	Kouloubi (Fè), Gouloubi (Nago)	Nago, Fè (Leaves)
52	*Pseudocedrela kotschyi*	Meliaceae	Asntélémr (Lamba)	Lamba (Bark)
53	*Psorospermum alternifolium*	Clusiaceae	Kpinon-kpinon (Fè)	Fè (Leaves)
54	*Raphionacme brownii *	Asclepiadaceae	Kousséligou (Gourmantché), Kohounsèhounta (Wama)	Gourmantché, Wama (Tuber)
55	*Rauvolfia vomitoria*	Apocynaceae	Essô èyèdjè (Fè)	Fè (Leaves)
56	*Saba comorensis*	Apocynaceae	Louou (Lamba)	Lamba (Fruits)
57	*Sarcocephalus latifolius*	Rubiaceae	Bounangnibou (Gourmantché), Athithélou (Lamba)	Lamba (Leaves, Roots, fruits); Gourmantché (Fruits)
58	*Sclerocarya birrea*	Anacardiaceae	Mounannikmon (Otamari), Bounanmag'bou (Gourmantché)	Otamari (Fruits, Leaves); Gourmantché (fruits)
59	*Sesamum radiatum*	Pedaliaceae	Dossé (Nago), Koumonkoun-adjagbalè (Fè), Ungangoun (Gourmantché), Natawourou (Lamba), Agbô (Mahi)	Mahi, Fè, Gourmantché, Nago, Lamba (Leaves)
60	*Solanum erianthum*	Solanaceae	Mon (Fè)	Fè (Leaves)
61	*Sterculia tragacantha *	Sterculiaceae	Akèmonkodjèko (Fè)	Fè (Leaves)
62	*Strychnos spinosa*	Loganiaceae	Fountoumdrô (Lamba)	Lamba (fruits and Roots)
63	*Talinum triangulare*	Portulacaceae	Odondon (Nago), Odondon (Fè), Glassoéman (Mahi)	Nago, Fè, Mahi (Leaves)
64	*Tamarindus indica *	Leguminosae	Boupouguibou/Boupouobou (Gourmantché), Timtélém (Lamba)	Gourmantché (Fruits, Leaves); Lamba (Fruits)
65	*Vernonia colorata*	Asteraceae	Arikoro (Nago)	Nago (Leaves)
66	*Vitellaria paradoxa *	Sapotaceae	Kotoblè (Mahi), Emin (Fè, Nago), Boussanbou (Gourmantché), Sèmou (Lamba)	Mahi, Fè, Nago, Lamba (fruits), Gourmantché (fruits, bark)
67	*Vitex doniana*	Verbenaceae	Bougaanbou (Gourmantché), Akpagnarou (Lamba), Fonman (Mahi), Ewa (Fè), Akoumanlapka (Nago)	Mahi, Fè, Gourmantché, Nago, Lamba (Leaves, fruits)
68	* Ximenia americana*	Oleracea	Klivovoé (Mahi), Boumirinbou (Gourmantché)	Mahi (fruits); Gourmantché (Fruits, Leaves, Roots)
69	*Zanthoxylum zanthoxyloides*	Rutaceae	Tchanouwèlè (Fè)	Fè (Leaves, Roots, Bark, Thorns)

**Table 2:** Number of plant species under domestication per village and their distribution per type of plant and by habitat.

Villages	Total	Types of plants			Habitat			
		Trees	Shrubs	Herbs	Forest	Fallow	Cultivated field	Home garden
Banon	33	8	4	21	4	5	7	2
Gbédé	22	6	2	14	10	12	8	3
Aglamidjodji	18	5	3	10	8	7	5	1
Korontière	27	14	6	7	8	7	6	3
Batia	21	12	3	6	10	8	7	3

**Table 3:** Knowledge of the species and of their biology by the local communities.

Ethnic groups	Total	Knowledge of the species		Knowledge of the species' biology		Period of availability		
		Widely known	Little known	Known	Unknown	AS	RS	DS
Ditamari/Lamba	27	17	10	18	9	8	16	3
Gourmantché	21	13	8	15	6	5	9	7
Mahi	18	12	6	10	8	5	11	2
Nago	36	28	8	27	9	9	25	2

*AS: all seasons, RS: rainy season, DS: dry season.*

**Table 4:** Domestication levels of the species and their variations across villages (species found only at Step 1 are not included).

Number	Scientific name	Domestication levels				
		Aglamidjodji	Banon	Batia	Gbédé	Korontière
1	*Adansonia digitata *	0	0	2	0	1
2	*Bixa orellana*	—	3	—	—	—
3	*Bombax costatum*	2	—	—	—	0
4	*Caesalpinea bonduc *	2	4	—	3	—
5	*Calotropis procera*	—	—	—	—	4
6	*Ceiba pentandra*	—	2	—	—	—
7	*Celosia argentea*	4	—	—	3	—
8	*Celosia trigyna*	—	3	—	3	—
9	*Ceratotheca sesamoides *	0	1	—	1	1
10	*Corchorus tridens*	—	—	—	3	—
11	*Clausena anisata*	—	1	—	—	—
12	*Cleome ciliata *	1	—	—	—	—
13	*Cleome gynandra *	—	—	—	2	—
14	*Crassocephalum rubens*	3	2	—	3	—
15	*Detarium microcarpum *	—	—	1	—	0
16	*Dioscorea praehensilis *	—	5	—	—	—
17	*Ficus abutilifolia *	—	2	—	1	—
18	*Ficus ingens*	—	—	1	—	—
19	*Haumaniastrum caeruleum*	—	2	—	—	—
20	*Hibiscus sabdariffa *	—	3	—	3	—
21	*Justicia tenella*	—	2	—	—	—
22	*Lagenaria siceraria *	—	—	—	3	—
23	*Launeae taraxacifolia*	2	2	—	2	—
24	*Lippia multiflora*	4	1	—	—	—
25	*Ocimum americanum*	—	3	—	—	—
26	*Ocimum basilicum*	0	1	—	2	—
27	*Ocimum gratissimum*	3	2	—	—	—
28	*Parkia biglobosa *	1	1	1	2	2
29	*Piliostigma thonningii *	—	—	—	—	1
30	*Platostoma africanum *	—	1	—	1	—
31	*Rauvolfia vomitoria*	—	2	—	—	—
32	*Sesamum radiatum*	2	2	4	1	0
33	*Solanum erianthum*	—	2	—	—	—
34	*Talinum triangulare*	1	2	—	2	—
35	*Tamarindus indica *	—	—	2	—	1
36	*Vernonia colorata*	—	—	—	1	—
37	*Vitellaria paradoxa *	1	1	2	1	2
38	*Vitex doniana*	1	1	1	1	0

**Table 5:** Contribution of some species under domestication to household income generation.

Species	Minimum (US$)	Maximum (US$)
*Caesalpinea bonduc*	7	8
*Celosia argentea*	100	140
*Celosia trigyna*	2	5
*Cochlospermum tinctorium*	20	144
*Ceratotheca sesamoides*	10	90
*Crassocephalum rubens*	3	10
*Dioscorea praehensilis*	9	30
*Haumaniastrum caeruleum*	4	8
*Launeae taraxacifolia*	120	192
*Lippia multiflora*	2	10
*Parkia biglobosa*	400	600
*Sesamum radiatum*	50	96

**Table 6:** Classification of the species under domestication according to the gender and to their specific utilization.

Group of species	Total	Ethnic groups				Type of plant				
		NA	MA	GO	LD	LV	NV	Fr	Tb	Md
Species being domesticated by women	31	20	09	04	04	22	02	02	00	05
Species being domesticated by men	18	06	03	09	16	02	01	09	02	04
Species being domesticated by both men and women	20	10	06	08	07	04	03	03	01	09

Total	69	36	18	21	27	28	06	14	03	18

*NA: Nago, MA: Mahi, GO: Gourmantché, LD: Lamba/Ditamari, LV: leafy vegetable, NV: nonleafy vegetable, Fr: fruit, Tb: Tuber crop, Md: medicinal plant.*
